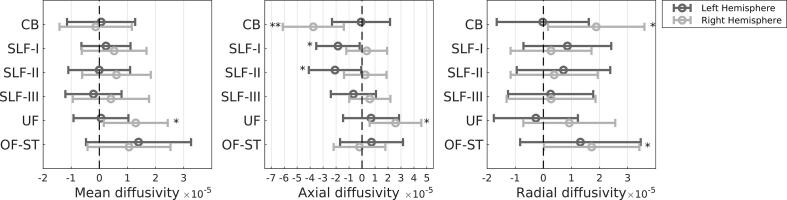# White matter diffusivity and its correlations to state measures of psychopathology in male refugees with posttraumatic stress disorder

**DOI:** 10.1016/j.nicl.2021.102929

**Published:** 2021-12-28

**Authors:** Sigurd Wiingaard Uldall, Henrik Lundell, William F.C. Baaré, Hartwig Roman Siebner, Egill Rostrup, Jessica Carlsson

**Affiliations:** a Competence Centre for Transcultural Psychiatry (CTP), Mental Health Centre, Ballerup, Denmark; b Danish Research Centre for Magnetic Resonance, Centre for Functional and Diagnostic Imaging and Research, Copenhagen University Hospital - Amager and Hvidovre, Copenhagen, Denmark; c Department for Neurology, Copenhagen University Hospital Bispebjerg, Copenhagen, Denmark; d Institute of Clinical Medicine, Faculty of Health and Medical Sciences, University of Copenhagen, Copenhagen, Denmark; e Center for Neuropsychiatric Schizophrenia Research and Center for Clinical Intervention and Neuropsychiatric Schizophrenia Research, Mental Health Centre, Glostrup, Copenhagen University Hospital, Denmark

**Keywords:** PTSD, Refugees, State measures, White matter, MRI, DWI, FA, DTI

## Abstract

Post-traumatic stress disorder (PTSD) is a heterogenous condition and the underlying neurobiology is still poorly understood. In this study, we tested the hypothesis that PTSD is associated with microstructural changes in white matter (WM) fibre tracts that connect regions involved in emotional processing, memory, attention, and language. Furthermore, we examined how different response patterns to individualized trauma-provoking stimuli related to underlying WM microstructure. Sixty-nine trauma-affected male refugees with PTSD (N = 38) or without PTSD (N = 31) underwent clinical assessments and diffusion-weighted magnetic resonance imaging (DWI) of the whole brain at 3 Tesla. Diffusion tensor metrics were computed from DWI data and used to characterize regional white-matter microstructure. An automated tract segmentation method was used to extract diffusion tensor metrics from subject-based reconstructions of tract segments (ROI), including uncinate fasciculus (UF), cingulum bundle (CB), superior longitudinal fasciculus (SLF) in three subdivisions (SLF I - III), and fibre bundles connecting orbito-frontal cortex to striatum (OF-ST). Outside the scanner we obtained measures of immediate (state) arousal, avoidance and dissociation symptoms assessed in response to auditory exposure to a personal traumatic memory. Using mean FA of the middle part of each ROI, mixed ANOVA revealed a significant interaction between group, ROI and hemisphere. Post-hoc comparisons showed that, relative to refugees without PTSD, refugees with PTSD had lower FA in right CB, left SLF-I, bilateral OF-ST and bilateral SLF-II. Mean FA scaled negatively with avoidance in right CB while mean FA in bilateral UF scaled positively with individual scores reflecting dissociation symptoms. The results support a pathophysiological model of PTSD that implicates limbic structures, prefrontal cortex and striatum. The results also emphasize the need to consider PTSD’s multifaceted manifestations when searching for functional-structural relationships.

## Highlights

• We investigated diffusivity of white matter fibre tracts in male refugees with PTSD.
• Tract-specific diffusion tensor metrics were extracted using an automated tract segmentation method.
• PTSD was associated with decreased fractional anisotropy (FA) in several tracts.
• State dependent measures of dissociations and avoidance scaled with FA.

## 1 Introduction

Post-traumatic stress disorder (PTSD) is a psychiatric condition that develops in response to an event of exceptionally threatening or catastrophic nature (American Psychiatric Association, 2013). Typical features of PTSD include episodes of repeated reliving of the trauma in intrusive memories (“flashbacks”) occurring against a persistent background of emotional blunting and avoidance of activities reminiscent of the trauma. Despite these commonalities, there are also large variations in the symptomatology of PTSD (Galatzer-Levy and Bryant, 2013), such as different emotional responses to traumatic memories and triggers. While most patients with PTSD become distressed and experience physical reactions of arousal (e.g. racing heart), some are more inclined to react with symptoms of dissociation (e.g. feeling disconnected from the body or like being in a fog) (American Psychiatric Association, 2013, van Huijstee and Vermetten, 2018). Also, while some recall traumatic memories in full bloom others, despite experiencing emotional distress, do not vividly recall the trauma i.e. have reduced trauma memory specificity (Hermans et al., 2005).

Structural and functional magnetic resonance imaging (MRI) studies have shown PTSD to be characterized by morphological and functional alterations, such as increased grey matter volume and increased activity in limbic and prefrontal structures associated to symptoms of dissociation and arousal, respectively (Bryant et al., 2008, Daniels et al., 2016, Lanius et al., 2010, Nardo et al., 2013). The neuroimaging findings led to a neurobiological “hyperinhibition” model of dissociative symptoms (van Huijstee and Vermetten, 2018). According to this model, prefrontal structures exert enhanced inhibitory influence on limbic regions, such as insula and amygdala, triggering a state of hypoemotionality, which is generally experienced as being in a dream, being disconnected from the body or as being in a fog. Conversely, PTSD symptoms characterized by arousal and fear are suggested to reflect the reverse neural state resulting from attenuated inhibitory prefrontal drive of limbic structures.

Moreover, studies employing diffusion-weighted magnetic resonance imaging (DWI), which is sensitive to the diffusion of water molecules, have probed for possible white matter deficits in PTSD. (Daniels et al., 2013, Siehl et al., 2018). DWI data have most frequently been analysed by applying the diffusion tensor imaging (DTI) framework (Basser et al., 1994) to assess white matter microstructure pathology and plasticity at the voxel level (Sampaio-Baptista and Johansen-Berg, 2017). One commonly derived measure is regional fractional anisotropy (FA) (Basser and Pierpaoli, 1996), which reflects the degree of water diffusion directionality and is a sensitive probe of cellular structure (O’Donnell and Westin, 2011). Decreasing FA values may reflect various pathological conditions such as a regional decrease in myelination, a reduction in axon density or degeneration of the intra-axonal space (Beaulieu, 2002, Le Bihan et al., 2001, Soares et al., 2013).

DWI studies have reported decreased FA related to PTSD in uncinate fasciculus (UF), cingulum bundle (CB) and superior longitudinal fasciculus (SLF) in PTSD (Averill et al., 2018, Daniels et al., 2013, Fani et al., 2012, Huang et al., 2012, Koch et al., 2017, O'Doherty et al., 2018, Schuff et al., 2011, Siehl et al., 2018, Zhang et al., 2012). Among others, these tracts connect prefrontal cortices with amygdala and insula (UF) (Schmahmann and Pandya, 2006, Von Der Heide et al., 2013), frontal with medial temporal, e.g. hippocampal, brain regions (CB) (Bubb et al., 2018, Schmahmann and Pandya, 2006) and prefrontal with parietal, temporal and occipital brain regions (SLF) (Makris et al., 20052005, Petrides and Pandya, 2009, Reislev et al., 2016). Reduced FA in these tracts suggest compromised structural connectivity which, in light of PTSD-symptomatology, concur with their general implication in top-down regulation of negative mood (UF) (Milad and Quirk, 2012), executive control and memory function (CB) (Bubb et al., 2018, Bubb et al., 2017) and higher cortical functions, such as attention, language and verbal learning (SLF) (Nakajima et al., 2020). However, the results of DWI studies are still equivocal (Dennis et al., 2019) with PTSD being associated with both increased and decreased FA, or not at all (Siehl et al., 2020, Siehl et al., 2018). Also, it is unclear if changes in WM occur across hemispheres or is predominantly lateralized (Daniels et al., 2013). Since investigations hitherto have studied predominantly single-trauma affected groups and war-veterans, it is also unclear to which degree the current findings may extent to trauma-affected patient groups at large, including trauma-affected refugees.

The present study aimed to assess the microstructure of WM fibre tracts of brain networks involved in emotional processing, memory, attention, and language, in treatment seeking refugees with PTSD using an automated tract segmentation method. We hypothesized that PTSD would be associated with decreased FA in CB, UF and SLF compared to a healthy control group of trauma-affected refugees (TRC). Also, since mood disturbances in PTSD have been linked to decreased activity in orbito-frontal (OF) cortex and ventral striatum (Felmingham et al., 2014, Frewen et al., 20112011, Frewen et al., 2010, Olson et al., 2018, Sailer et al., 2008) we also hypothesized decreased FA in fibre tracts connecting striatum to OF (OF-ST). Additionally, we investigated different response patterns to individual trauma provoking stimuli in PTSD and their relations to specific alterations in the microstructure of CB and UF. Pursuing a theory based on predominantly fMRI data, we hypothesized that FA in UF would be negatively associated with arousal symptoms but positively associated with dissociative symptoms, reflecting hypo- and hyperinhibition of limbic regions from mPFC, respectively (van Huijstee and Vermetten, 2018). Moreover, given that CB is involved in memory functions (Bubb et al., 2018) and FA measures in CB have been found to be negatively associated with trait measures of avoidance in PTSD (O'Doherty et al., 2018), we expected that FA in CB would be negatively correlated with state measure of avoidance.

## 2 Study cohort and behavioural and clinical assessments

### 2.1 Participants

All participants were trauma-affected male refugees or male family members reunified to a refugee. The inclusion of only male was motivated by another component of the project concerning comparisons of volumetric MR data between refugees and military veterans (mainly males). Seventy-eight participants were included from May 2016 – April 2018. The sample size was initially determined to answer a research question related to an fMRI component of the study testing a salience contrast in a reward paradigm. Here, the sample provided a statistical power of 80% at an alpha level of 0.05 for observing a standardized difference of 1.23 (Uldall et al., 2020b). Although the sample size in the present paper was not determined by a power calculation related to DTI analyses, previous DTI studies comparing PTSD and healthy controls on FA have been able to show a statistically significant effect with considerable smaller samples (see Siehl et al., 2018 for a review). Participants with PTSD were recruited at the Competence Centre for Transcultural Psychiatry (CTP), Ballerup, Denmark, where multidisciplinary services are provided to trauma-affected refugees without a primary psychotic or bipolar disorder (Carlsson et al., 2014). All PTSD participants were enrolled in a treatment program at CTP consisting of 24 sessions, which included prolonged exposure therapy. Trauma-affected refugee controls (TRC) were recruited via advertisements (public posters and on the internet) and from family and acquaintances of interpreters at CTP and matched to PTSD participants for country of origin (but not for lifetime trauma experience). An attempt was also made to match the two groups for age but due to limited recruitment possibilities this was not fully achieved. Participants were primarily from Syria (29%), Iraq (24%), Afghanistan (21%) and Iran (9%). Yemen, Bosnia, Lebanon, South Sudan, Egypt, Turkey and Jordan were also represented.

None of the participants with PTSD had received antipsychotic medicine within the last month, but antidepressants were not an exclusion criterion. Also, psychiatric symptoms before the onset of PTSD was an exclusion criterion. For all participants, alcohol and substance abuse and moderate or severe brain injury were exclusion criteria and all participants underwent a substance abuse urine test (Rapid Response, BTNX Inc., Canada) and completed the Alcohol, Smoking and Substance Involvement Screening (Ali et al., 2002). Psychotropic medication was an exclusion criterion for TRC. Traumatic brain injury (TBI) was identified using the Ohio State University Identification Method (Corrigan and Bogner, 2007) where mild TBI includes report of brain or neck trauma followed immediately by being dazed, having memory lapses or loss of consciousness for<30 min. MRI exclusion criteria included claustrophobia and standard MRI safety incompatibility.

The study was approved by the Danish Ethical Committee of Science (H-15006293) and the Danish Data Protection Agency (2012–58-0004) and conducted in accordance with the declaration of Helsinki. All participants gave written informed consent after written and oral explanation of study aims and procedures and were compensated with a small fee in addition to reimbursements of public transportation. All participants were part of two other studies investigating the reward circuitry (Uldall et al., 2020b) and the relationship between symptom provocation and emotional processing (Uldall et al., 2020a) with functional MRI.

### 2.2 Clinical assessment

All questionnaires were available in the participants’ native language and translators were accessible throughout the study. The Schedules for Clinical Assessment in Neuropsychiatry (SCAN) interview was used to assess the presence of PTSD, depression and enduring personality change in all participants (Wing et al., 1990). SCAN was also used to exclude participants with manic episodes or schizophrenia. Participants with PTSD were further interviewed with the Clinician-Administered PTSD Scale for DSM-5 (CAPS, Weathers et al., 2013), assessed for the past month. The CAPS provide a measure of symptoms of intrusion, avoidance, arousal, mood, reactivity and cognition. All participants were given the Life Event Checklist (LEC, Weathers et al., 2013). The LEC assesses exposure to 16 different types of potentially traumatic events and the number of experienced events was used as outcome score. Participants were inquired about their trauma, medical, social and smoking history.

### 2.3 Measures of arousal, avoidance and dissociation in response to trauma provocation

State symptoms of arousal, avoidance and dissociation were measured on the same day as the DWI data was collected in response to audible exposure to a personal traumatic memory. To this end participants, inside the MR-scanner, listened to a 30 sec long personal traumatic memory narrated in second person singular by either a translator or the first author (SWU). Participants were subsequently asked to recall the memory as vividly as possible for additional 30 sec. Heart rate was monitored before and after the script using a pulse oximetry probe attached to the right index finger for the duration of the scanning session. Heart rate was expressed as mean beats per minute. The procedure was identical to previous published methods (Hopper et al., 2007, Shin et al., 1997). The script depicted vivid descriptions of ambient and sensory experiences from a well-remembered past traumatic memory and had been composed on a previous day by the participants and the first author (SWU). After symptom provocation, participants were scanned with functional MRI (fMRI) to investigate a research question of another component of the project published elsewhere (Uldall et al., 2020a). After the fMRI session participants were interviewed with the Script-Driven Imagery Scale (RSDI) (Hopper et al., 2007) that captures symptoms of *arousal*, *avoidance* and *dissociation* in response to symptom provocation. The RSDI uses four questions to assert symptoms of dissociation and arousal and three questions to assert avoidance. The interview took place directly after the scanner session and the participant was carefully instructed to report symptoms severity as experienced in immediate response to the script (i.e. while inside the scanner). Notably, we followed the same procedure as used in previous studies under supervision to creators of the RSDI (Hopper et al., 2007). Each question is answered on a 7-point Likert scale (0 = *”not at all”*, 6 = *”a great deal”*) and for each symptom cluster the answers are summed to subscores. These subscores are here used as state dependent measures.

## 3 Magnetic resonance imaging

### 3.1 Data acquisition

All participants were scanned at the Danish Research Centre for Magnetic Resonance using a 3 T Philips Achieva MRI system (Philips Healthcare, Best, The Netherlands) with a 32-channel head receive coil and body transmit coil. Whole brain DWI data was collected using a pulsed gradient spin echo sequence with diffusion weighting b = 1000 s/mm^2^ (gradient amplitude = 62 mT/m, duration = 12.5 ms and separation = 27.5 ms) in 62 unique and uniformly distributed gradient directions created from an electrostatic simulation. The gradient vectors where distributed over two hemispheres to evenly sample eddy current distortions. Image readout was performed with echo planar imaging (EPI) with 2^3^ mm^3^ isotropic resolution (TR = 10,259 ms, TE = 55 ms, SENSE parallel imaging factor = 2, partial Fourier factor = 0.695, Posterior-Anterior (PA) phase encoding direction). Additional b = 0 scans were performed with the phase encoding in the original PA and reversed Anterior-Posterior (AP) directions with five repetitions for each condition creating pairs of images with field induced geometrical distortions in opposite directions but with the same magnitude.

The scan session also included a structural protocol for radiological evaluation with the following imaging sequences: 3D T1-weighted MPRAGE (TR/TE = 6.0 ms/2.7 ms, inversion time = 1000 ms, sagittal slices, isotropic 0.85^3^ mm^3^ voxels, flip angle = 8°); 3D T2-weighted (TR/TE = 2500 ms/250 ms, isotropic 0.85^3^ mm^3^ voxels, flip angle = 90°); 2D T2-weighted (TR/TE = 3381/80 ms, axial slice orientation with in plane resolution 0.45^2^ mm^2^ and slice thickness 3 mm and 1 mm slice gap, flip angle 90°); 3D Fluid-attenuated inversion recovery (FLAIR) (TR/TE = 4800 ms/327 ms, TI = 1650 ms, isotropic 1^3^ mm^3^ voxels, flip angle 90°); and a 3D susceptibility weighted gradient echo image (TR/TE = 27/20 ms, anisotropic voxels (FH 1.5 mm AP 0.76 mm RL 0.76 mm, flip angle 20°).

### 3.2 DWI processing pipeline

DWI data were preprocessed to correct for motion and image distortions from eddy currents and field inhomogeneities using the eddy and topup tools in FSL5.0.9 (http://fsl.fmrib.ox.ac.uk/) (Andersson et al., 2003, Andersson and Sotiropoulos, 2016). The eddy tool estimates eddy current distortions and motion across different diffusion encoding gradient directions and topup calculates the distortion fields from the EPI-acquisitions with opposed phase encoding directions. DWI data were finally rigid body registered to the FSL FA MNI template with FSL/FLIRT, using an intermediate FA image calculated from the corrected dataset as source. Finally, the original data was resliced to 2^3^ mm^3^ resolution in MNI space with the combined transformations calculated in previous steps. All raw data and derived maps were visually assessed for quality issues and artefacts.

Tract based segmentation was performed on the preprocessed DWI data in MNI space with the TractSeg toolbox (version 1.7.1 retrieved from https://github.com/MIC-DKFZ/TractSeg) extending on the MRTRIX3 software package (http://www.mrtrix.org) (Tournier et al., 2019, Wasserthal et al., 2018). In short, TractSeg uses constrained spherical deconvolution (CSD) (Tournier et al., 2008) to extract peak maps with up to three dominant fibre directions in each voxel and utilizes a deep learning approach to cluster coherent tracts directly on the peak maps without streamline generation (Wasserthal et al., 2018). The method segments and labels regions based on a pre-trained database of 72 tract systems manually dissected with tractography in 105 subjects from the high quality Human Connectome project database (Van Essen et al., 2013). Output data consist of masks covering the individual fibre tracts and labelled as either beginnings or endings. We produced 10,000 probabilistic streamlines from the CSD peaks from the beginning of the mask (seed region) and excluded streamlines leaving the tract mask or not reaching the ending mask for each tract system of interest. The diffusion tensor (DT) and derived FA, mean diffusivity (MD), axial diffusivity (AD) and radial diffusivity (RD) metrics were computed using weighted least squares fitting using the dtifit tool in FSL.

**Fig. 1:**
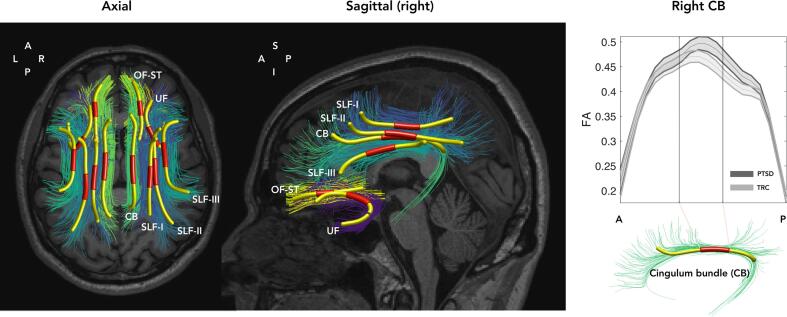
Tract based segmentation and estimation of DT metrics. **Left and middle panel**: Fibre tract bundles of interest, overlaid on a structural T1-weighted MPRAGE image, are presented for one subject in axial and sagittal projections (only tracts on the right side are shown for the latter). Tractography streamlines representing individual fibre bundles are shown as differently coloured thin lines. The centre lines of uncinate fasciculus (UF), orbitofrontal-striatal (OF-ST), superior longitudinal fasciculi (SLF I-III) and cingulum bundle (CB) are shown as thick yellow lines with the mid-segment used in the analysis highlighted in red. **Right:** The mean FA (fractional anisotropy) profiles along the tracts (solid lines) and 95% confidence intervals (shaded) for the PTSD and TRC groups extracted from CB with the section between the two vertical lines representing the mid-segment. Mean FA as well as mean, radial and axial diffusivities (MD, RD, AD) for the mid-segments of the fibre tracts of interest were used in the statistical analyses. (A, P, S, I, R and L denote anterior, posterior, superior, inferior, left and right directions.) (For interpretation of the references to colour in this figure legend, the reader is referred to the web version of this article.)

**Fig. 2:**
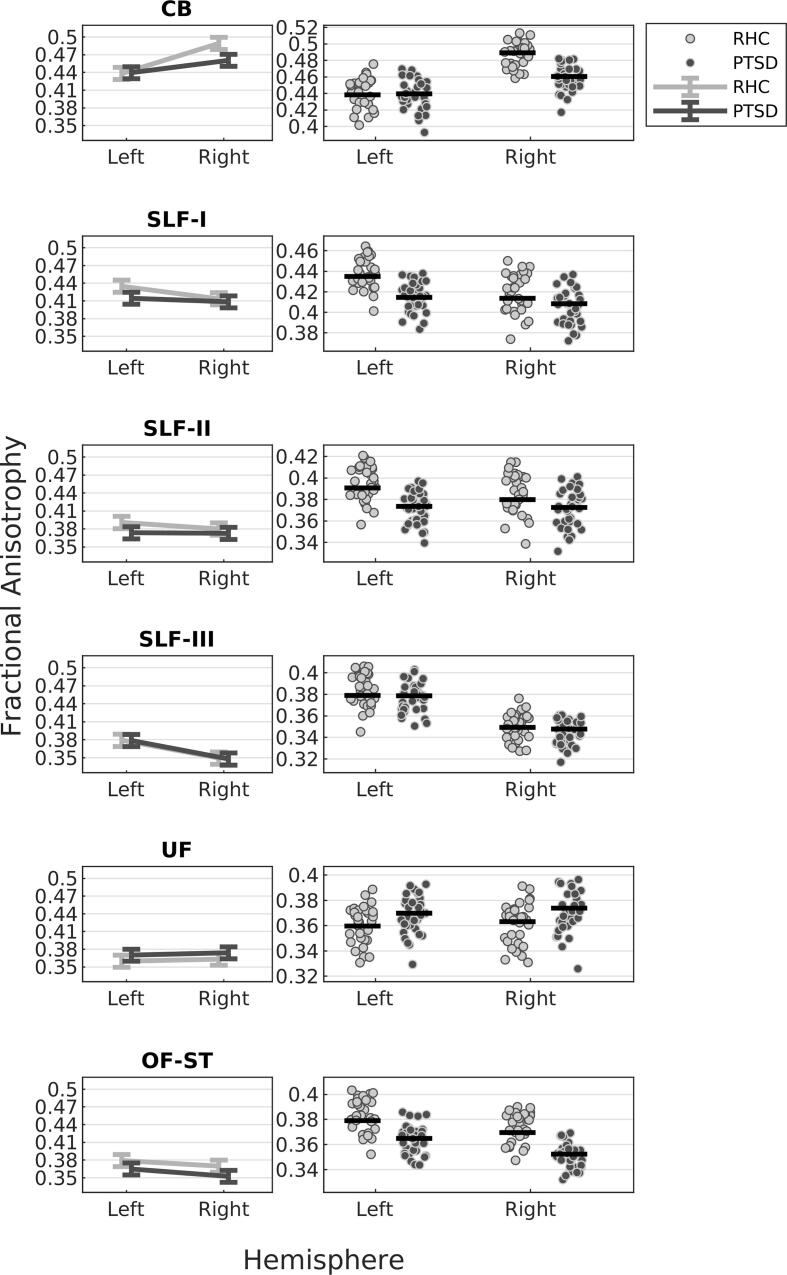
Visualization of the individual tract data underlying the significant Group × Tract × Hemisphere interaction, separate for TRC and PTSD and for left and right tract segments. Left panels: covariate-adjusted mean FA and 95% confidence interval (vertical lines). Right panels: covariate-adjusted individual data points and covariate-adjusted mean FA (horizontal lines)*.*The values are derived from a mixed ANOVA model with tract FA as the dependent measure, group as between-subject variable, tract and hemisphere as within-subject variables and age, previous mild traumatic brain injury, whole brain FA and tract volume as covariates. CB = Cingulum Bundle, SLF-I, SLF-II and SLF-III = Superior Longitudinal Fasciculus, segments I-III, UF = Uncinate Fasciculus, OF-ST = Orbitofrontal-Striatum, tract segments = Fibres of interest.

**Supplementary figure 1:**
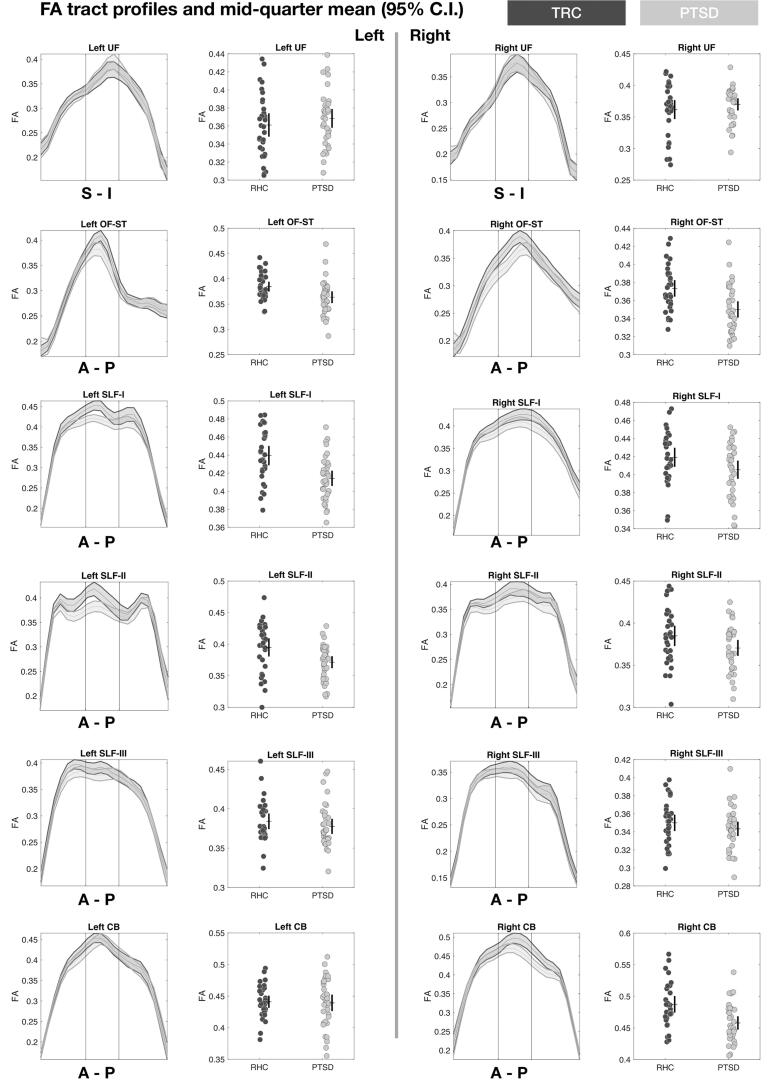


FA, MD, AD and RD profiles were extracted at 20 nodes along tracts using the Tractometry method proposed by Yeatman et al (Yeatman et al., 2012) as implemented within the TractSeg framework. Tract profiles where calculated as the mean at each node excluding points deviating five standard deviations in distance along or four standard deviations in distance perpendicular from a Gaussian distribution of the nodes’ coordinates as proposed by the original method (Yeatman et al., 2012) (Fig. 2). Mean FA, MD, AD and RD were calculated from the mid-segment of the tract profiles reflecting one quarter of its length (point 8 to 12 out of 20) of each of the six fiber tracts of interest (tract segments), including UF, CB, OF-ST and the three SLF I-III segments, separately for left and right hemisphere. Mid-segments were used to exclude possible partial volume effects with cortex, intersecting pathways and to minimise possible fibre dispersion effects on the DT metrics towards the tract endings (see whole tract profiles for the right CB in Fig. 1 and Inline Supplementary Figure 1 for all tracts). For brevity FA/MD/RD/AD (and the categorical variable Tract) will henceforth refer to tract segment mean FA/MD/RD/AD, respectively. Since the DT metrics is also sensitive to partial volume effects modulated by the overall tract size (Vos et al., 2011), we extracted the volume of each fibre tract mask as an additional covariate. The metrics from the mean of all 72 tract mid-segments were computed as a covariate to correct for global white matter changes extracted on par with our FOI.

### 3.3 DTI measures

FA was our main measure of interest. FA reflects the degree of water diffusion directionality in a voxel and captures combined effects in axial diffusivity (AD), parallel to the axon axis, and radial diffusivity (RD), perpendicular to axon axis. An increase in FA is associated with a disproportionate decrease in RD relative to AD or vice versa a disproportionate increase in AD relative to RD. We extracted AD and RD to explore the nature of any observed effects for FA. These measures can be modulated by several factors such as overall alignment of axons on a voxel level and partial volumes of more free water compartments, down to microstructural changes. At the microstructural level, RD and AD can increase by decreased density or myelination of axons, providing more mobility in the extra-axonal space. Moreover, demyelination may further decrease RD as this increases water mobility across the cell membrane. AD may decrease with degeneration of the cytoskeleton and connected changes to cytosol viscosity or variations in diameter along the axon. Additionally, we extracted mean diffusivity (MD = (AD + 2·RD)/3), a measure of the magnitude of water diffusion in a voxel disregarding directional information. MD reflects the overall density and permeability of tissue and is negatively proportional to the amount of cell membranes and cellular structures (Beaulieu, 2002; D Le Bihan et al., 2001, Soares et al., 2013).

### 3.4 Radiological assessment of structural data and quality assurance of DWI data

All structural clinical scans were evaluated for clinical abnormalities by two radiologists, and participants were informed of the results of the evaluation. Eleven participants (7 participants with PTSD and 4 TRC) had minor pathological findings such as unspecific gliosis, partially empty sella turcica and leukoaraiosis. Five participants (2 participants with PTSD, 3 TRC) had an additional clinical scan due to suspicion of more serious pathology. In two cases this led to referrals to the neurosurgical department of Rigshospitalet, Copenhagen, Denmark, for evaluation of a small focal intracerebral process and cavernous haemangioma. In the former case the process was not evaluated to be pathological and in the latter the patient was successfully operated. One participant with PTSD presented with widespread ischemic changes in the diffusion-weighted image and was excluded from further analyses. Two additional participants with PTSD were excluded due to respectively excessive motion artefacts and a wrongly set field of view.

## 4 Statistics

Tract segment DT metrics, behavioural, psychopathological and demographic data were analysed with the statistical software R (R Development Core Team, 2020). For each participant there were 48 outcome measures; mean FA, MD, AD and RD from the mid-segment of the left and right hemisphere from CB, UF, SLF I, SLF II, SLF III, OF-ST. To test our hypotheses, we used FA as the primary dependent variable. Furthermore, as the different DT metrics complement each other in interpretation of the microstructure of white matter fibre tracts (Alexander et al., 2007), we investigated MD, AD and RD in follow-up exploratory analyses using the same statistical approach as detailed below for FA. All DT metrics were tested for the assumption of normal distribution with the Kolmogorov-Smirnov test (Massey, 1951) and for outliers using Grubbs’s test (Hodge and Austin, 2004). The threshold for significance was set to α = 0.05.

### 4.1 An omnibus model for testing group differences in fibre tract FA

To test group differences in tract segments FA, we used mixed analysis of variance (m-ANOVA). We fitted a model with group as between-subject variable (PTSD, TRC) and Tract (CB, UF, SLF I, SLF II, SLF III, FO-ST) and Hemisphere (left, right) as within-subject variables. Age, previous mild traumatic brain injury (mTBI: Yes, No) and whole brain mean FA were added as covariates, as these factors might effect DT metrics of individual tracts (Narayana, 2017, Sexton et al., 2014, Szczepankiewicz et al., 2013, Takao et al., 2011, Vos et al., 2011). False positive error rates related to violation of the assumption of sphericity were tested and corrected for, using respectively Mauchly’s test and Huynh-Feldt correction (Huynh and Feldt, 1976, Mauchly, 2007). The a priori contrasts of interests were the effects including Group i.e. main effect of Group and the Group × Tract and Group × Tract × Hemisphere interactions.

## 5 Planned follow-up analyses of tracts showing significant group effects

For fibre tracts showing group differences in FA, we used linear regression, correcting for the effects of age, mTBI and whole brain mean FA, to examine the possible effects of tract volume or number of personally experienced lifetime traumatic events. Furthermore, within PTSD participants we investigated for possible associations with psychotropic medication (Yes, No).

### 5.1 Testing the associations between arousal, dissociation and avoidance and FA in UF and CB

Mixed ANOVAs were used to test the hypotheses that (1) UF FA would be positively associated with state measures of dissociation and (2) negatively with state measures of arousal and that (3) CB FA would be negatively associated with state measures of avoidance. To this end we fitted a model to UF or CB FA with State Measure as an independent variable of interest and Hemisphere (left, right) as within-subject variable. Since we tested independent hypotheses for UF and CB, analyses across the two tracts were not adjusted for multiple comparisons. However, the two analyses concerning UF (hypothesis 1 and 2) were not independent. Consequently, we used a Bonferroni corrected alpha level of 0.025 (α = 0.05/2) to determine significance (Bonferroni, 1936). Age, mTBI and whole brain mean FA were entered as covariates. The contrasts of interest were the main effect of State Measure and the State Measure × Hemisphere interaction. Subsequently, we exploratively investigated the possible effect of Group (PTSD, TRC), and Group × State Measure and Group × State Measure × Hemisphere interactions. Given their exploratory nature, these tests were not corrected for multiple comparisons.

## 6 Results

### 6.1 Participants

One PTSD and two TRC participants withdrew consent due to a change of mind, one PTSD participant was later diagnosed with a primary psychotic disorder, three PTSD participants and one TRC opted out of the study due to anxiety during scanning and one TRC participant could not be scanned due to obesity. Hence, the study sample for which both clinical and MRI data was available consisted of 38 participants with PTSD and 31 TRC participants without PTSD or any another psychiatric diagnosis. Of these, three additional PTSD participants were excluded after evaluation of the structural clinical MRI scans and quality assurance of DWI data. Thus, the final study sample consisted of 35 PTSD participants and 31 TRC. The values of the clinical and demographical continuous variables of the 6 PTSD and 3 TRC participants that did not complete the study all fell within 1 standard deviation from the mean of PTSD and TRC participants that did complete the study.

Table 1 presents sociodemographic and clinical variables for all participants. Compared to TRC, PTSD participants were older, smoked more, had a lower educational level, and had experienced more traumatic events. Torture (48%), combat or exposure to a war zone (28%) and witnessing sudden violent death (20%) were marked as the most traumatic events among PTSD participants. In TRC these were combat or exposure to a war zone (32%), witnessing sudden violent death (23%) and physical assault (19%). PTSD participants scored higher on trait and state measures of arousal, avoidance and dissociation symptom severity than TRC. The mean heart rate for PTSD participants increased from 101 (SD = 13) to 105 (SD = 14) following the trauma script, whereas TRC participants heart rate was similar in both conditions (mean = 98; SD = 13). A repeated ANOVA test showed a non-significant main effect of group (F_(1,59)_ = 2.37, p = 0.129) but a significant group × script condition effect (F_(1,59)_ = 6.11, p = 0.016). The relative high heart rates in both groups and across script conditions are likely the result of the novel circumstances (awaiting listening to a past memory while lying in a MR scanner) under which heart rates were measured. Table 2 presents clinical characteristics of PTSD participants.

**Table 1:** Demographic and Clinical characteristics (n = 66).

Characteristics	PTSD (n = 35)	TRC (n = 31)	Group differences
Age^1^	44 (10)	38 (11)	**t_64_ = 2.54, p = 0.014**
Years in Denmark^1^	15 (10)	15 (10)	t_64_ = 0.15, p = 0.885
Smokers^2^	19 (54 %)	9 (29 %)	**χ^2^(1) = 4.29, p = 0.038**
Years of education^1^	13 (5)	16 (3)	**t_64_ = 2.43, p = 0.017**
Mild Traumatic Brain Injury ^2, a^	28 (80 %)	23 (74 %)	χ^2^(1) = 0.32, p = 0.574
Age at first traumatic event ^1^	18 (8)	17 (9)	t_64_ = 0.59, p = 0.559
Life Event Checklist ^3, b^	9 (7–11)	4 (1 – 6.75)	**t_64_ = 6.14, p < 0.001**
Symptoms of Arousal (state measure) ^1, c^	17.5 (4.3)	8.3 (5.6)	**t_64_ = 7.52, p < 0.001**
Symptoms of Avoidance (state measure) ^1, c^	7.8 (6.2)	3.6 (4.2)	**t_64_ = 3.16, p = 0.002**
Symptoms of Dissociation (state measure) ^1, c^	7.8 (6.4)	3.5 (4.4)	**t_64_ = 3.13, p = 0.003**

1 = mean, standard deviation in parenthesis

2 = number of participants, percentage in parenthesis

3 = median, interquartile range in parenthesis

^a^ includes report of brain or neck trauma followed immediately by being dazed, having memory lapses or loss of consciousness for less than 30 min.

^b^ Number of different kinds of traumatic events that either “happened to me” or were witnessed as defined by the Life Event Checklist-5.

^c^ In response to script-driven imagery of a traumatic memory. Measured with the Response to Script-Driven Imagery interview(Hopper et al., 2007)

**Table 2:** Clinical characteristics of PTSD participants (n = 35).

Duration of PTSD in years^1^	14 (10)	
Psychiatric co-morbidity^2^	30 (86 %)	
	Mild depression^3^	5 (17 %)
	Moderate depression^3^	17 (57 %)
	Severe depression^3^	7 (23 %)
	Periodic depression^3^	3 (43 %)
	Enduring personality change after catastrophic experience	13 (37 %)
Psychotropic medicine^2^	27 (77 %)	
	SSRI, No ^4^	12 (44 %)
	mg dose ^1^	104 (44)
	SNRI, No ^4^	3 (11 %)
	mg dose ^1^	113 (50)
	TeCA, No ^4^	20 (74 %)
	mg dose ^1^	13 (11)
	TCA, No ^4^	3 (11 %)
	mg dose ^1^	30 (15)
Clinician Administrated PTSD scale for DSM-5		
	Intrusion symptoms^1^	14.5 (4.1)
	Avoidance symptoms^1^	6.0 (1.8)
	Cognition and mood symptoms^1^	13.8 (3.5)
	Arousal and reactivity symptoms^1^	16.2 (5.1)

1 = mean, standard deviation in parenthesis

2 = number of participants, percentage in parenthesis, denominator = 35

3 = number of participants, percentage in parenthesis; denominator = 30

4 = number of participants, percentage in parenthesis; denominator = 27

SSRI = Selective serotonin reuptake inhibitor

SNRI = Serotonin-norepinephrine reuptake inhibitor

TeCA = Tetracyclic antidepressant

TCA = Tricyclic antidepressant

### 6.2 Quality assessment of tract segment DT data

All tract segments were consistently traced in all included participants and all outcome measures, i.e. left and right tract segments FA/MD/AD/RD, were normally distributed. In Inline Supplementary Figure 1, the raw tract segments FA data are visualized in scatter plots for each group and hemisphere. Based on Grubbs’s test (Hodge and Austin, 2004), the volume of right SLF-I in one PTSD participant was marked as a statistical outlier. The participant had normal appearing WM and running the analyses with or without this outlier did not give different results. Results below are presented with the outlier included.

### 6.3 Group differences in tract segments FA

The m-ANOVA showed a main effect of Group (F_(1,61)_ = 5.90, p = 0.018), a Group × Tract interaction (F_(5,305)_ = 3.26, p = 0.012, Huynh-Feldt corrected), and a Group × Tract × Hemisphere interaction (F_(5,305)_ = 5.23, p < 0.001). Furthermore, for the covariates we observed a significant effect of whole brain mean FA (F_(1,61)_ = 75.13, p < 0.001) but not of age (p = 0.482) or mTBI (p = 0.406). When additionally controlling for smoking, all three contrasts remained statistically significant (p-value range; 0.027–0.0004). The same was true when additionally, but separately, controlling for years of education (p-value range; 0.040 – 0.004), except for the Group × Tract interaction (F_(5,305)_ = 2.19, p = 0.069, Huynh-Feldt corrected).

**Supplementary figure 2:**
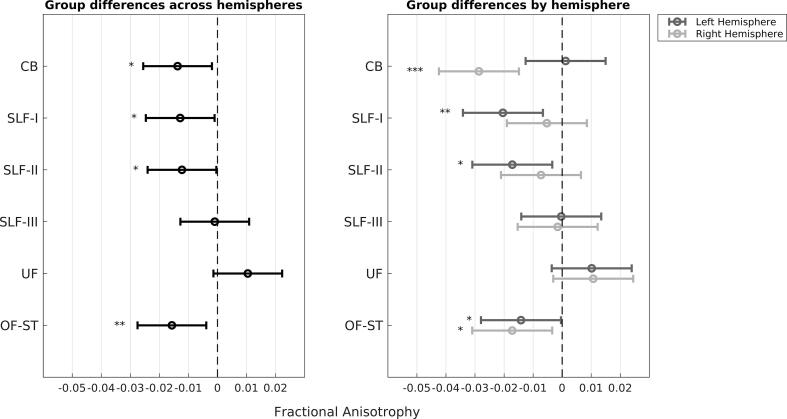


To interpret the observed 3-way interaction, we plotted for each tract segments the covariate-adjusted mean left and right hemisphere FA for each group (Fig. 2). Generally, except for SLF-III and UF, PTSD participants displayed lower FA than TRC. The significant Group × Tract × Hemisphere interaction indicates that groups differ as a function of tract and hemisphere. From Fig. 2, it can be seen that this especially may be the case for CB, SLF-I and SLF-II FA. PTSD participants, as compared to TRC, displayed lower right but comparable left CB FA. In contrast, PTSD participants displayed lower left but comparable right SLF-I and SLF-II FA. Additionally, PTSD participants, compared to TRC, displayed similar SLF-III, higher UF, and lower OF-ST FA. Follow-up analyses showed a significant Group × Hemisphere interaction for CB (F_(1,348)_ = 17.21, p < 0.001) and SLF-I (F_(1,348)_ = 4.43, p = 0.036) but not for SLF-II (p = 0.171). Furthermore, *t*-tests comparing groups on covariate-adjusted mean FA of right CB and left SLF-I respectively, revealed that both hemispheric differences were significant (right CB FA: t_561_ = 4.09, p < 0.001; left SLF-I FA: t_561_ = 2.91, p = 0.004). Finally, *t*-tests comparing groups on covariate-adjusted OF-ST, SLF-II and UF mean FA across hemispheres, revealed that group differences were significant for OF-ST FA (t_343_ = 2.61, p = 0.009) and SLF-II (t_343_ = 2.03, p = 0.042) but not UF (t_343_ = -1.73, p = 0.083). Covariate-adjusted mean FA for bilateral and unilateral tract segments, are visualized in Inline Supplementary Figure 2, together with results of pairwise *t*-tests comparisons.

### 6.4 Associations between mean tract segments FA and total tract volume, traumatic events and psychotropic medication.

**Supplementary figure 3:**
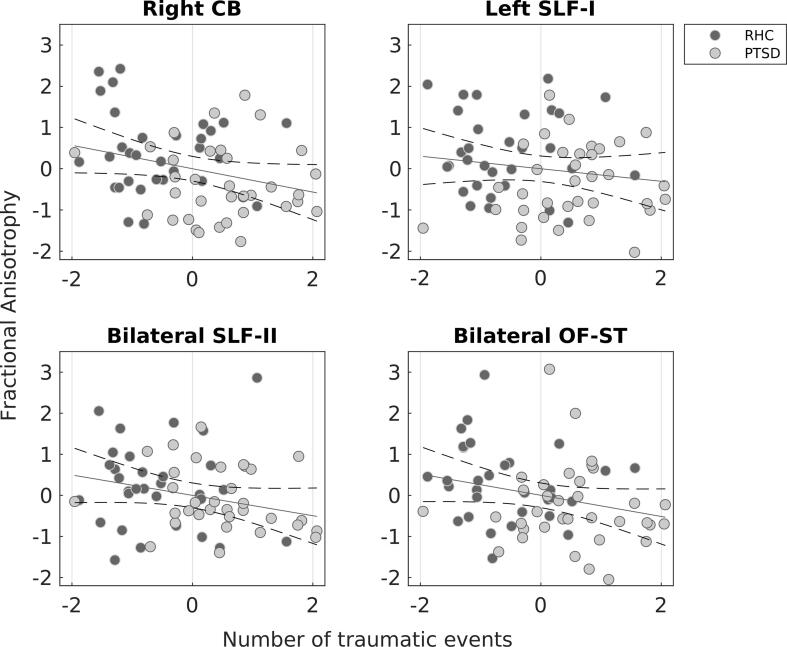


The exploratory linear regression analyses were restricted to tract segments showing group differences (right CB, left SLF-I and mean bilateral SLF-II and OF-ST). In each analyses the effects of age, mTBI and whole brain mean FA were controlled for. Due to their exploratory nature, the tests were not corrected for multiple comparisons. Total tract volume was not associated to tract FA in any of the analyses (p-values range: 0.057–0.762). More lifetime personally experienced traumatizing events was associated with lower right CB FA (β_standardized_ = -0.26, t_(61)_ = -2.30, p = 0.024) and mean bilateral SLF-II FA (β_standardized_ = -0.21, t_(61)_ = -2.06, p = 0.043) and OF-ST (β_standardized_ = -0.23, t_(61)_ = -2.10, p = 0.041). For left SLF-I the association was not significant (β_standardized_ = -0.12, t_(61)_ = -1.17, p = 0.248). The associations are visualized in Inline Supplementary Figure 3. When adding Group, the regression analyses did not show any Group × Trauma interactions (p-values range: 0.557–0.912). Finally, within PTSD participants, we did not observe any associations between right CB, left SLF-I, bilateral OF-ST or bilateral SLF-II and psychotropic medication use (p-values range: 0.202 – 0.986).

### 6.5 Post hoc testing for group differences in MD, AD and RD

For MD, we found no main effect of Group (F_(1,61)_ = 1.08, p = 0.301), or any interactions with Group (Group × Tract : F_(5,305)_ = 1.59, p = 0.16, Huynh-Feldt corrected; Group × Tract × Hemisphere: F_(5,305)_ = 1.19, p = 0.309). Likewise, we found no main effect of Group for AD (F_(1,61)_ = 0.31, p = 0.581). However, we did observe a Group × Tract (F_(5,305)_ = 2.98, p = 0.017, Huynh-Feldt corrected) and a Group × Tract × Hemisphere interaction (F_(5,305)_ = 6.26, p < 0.001). Visualizing left and right hemisphere AD for each group and each tract segments, showed that for CB, SLF-I and SLF-II PTSD participants displayed lower AD than TRC (Inline Supplementary Figure 4). Also, similar as we observed for FA, PTSD participants, as compared to TRC, displayed lower right but comparable left CB AD and lower left but comparable right SLF-I and SLF-II AD. For RD, we found no main effect of Group (F_(1,61)_ = 2.01, p = 0.161), and no Group × Tract (F_(5,305)_ = 0.67, p = 0.618, Huynh-Feldt corrected) or Group × Tract × Hemisphere (F_(5,305)_ = 1.67, p = 0.167) interaction. Covariate-adjusted mean left and right tract segments MD, AD and RD for both groups are shown in Inline Supplementary Figure 4.

### 6.6 Association between symptoms of arousal and UF FA

In contrast to our expectations, higher levels of arousal in response to the script-driven imagery were not associated with lower UF FA (Main effect arousal: F_(1,61)_ = 0.989, p = 0.324); Arousal × Hemisphere interaction: F_(1,61)_ = 0.06, p = 0.809). To investigate if the association between arousal and UF FA differed between PTSD participants and TRC, we added Group as a between-subject variable. No Group × Arousal (F_(1,59)_ = 0.34, p = 0.540) or Group × Arousal × Hemisphere (F_(1,59)_ = 1.652, p = 0.204) interactions were observed.

### 6.7 Association between symptoms of dissociation and UF FA

**Fig. 3:**
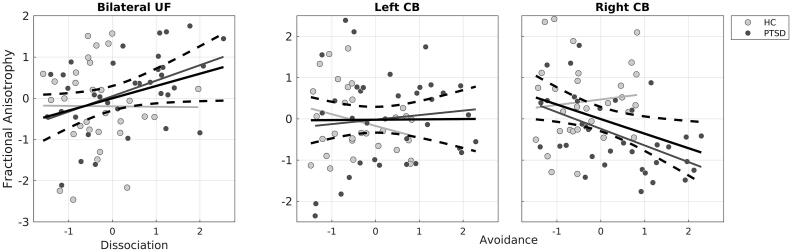
**Left panel;** Associations between symptoms of dissociation and bilateral UF FA (Across participants, p = 0.017, PTSD participants; p = 0.008;). **Middle and right panel;** Associations between symptoms of avoidance and CB FA separate for each hemisphere (Right CB: across participants; p = 0.004, PTSD participants; p = 0.009). The units are standardized residuals derived from linear regression models where the effect of age, mild TBI and whole brain FA have been controlled for. Solid lines represent the least square regression line across all participants (thick black lines and with 95 % confidence interval as dotted lines) and for TRC (light grey) and PTSD (dark grey). UF = Uncinate Fasciculus CB = Cingulum Bundle.

As hypothesized, we found a main effect of Dissociation on mean FA in UF (F_(1,61)_ = 6.04, p = 0.017), with higher levels of dissociation being associated with increased FA (see Fig. 3). This effect was statistically significant after adjusting for multiple comparisons using the Bonferroni method (corrected α = 0.025). We did not observe a Dissociation × Hemisphere interaction (F_(1,59)_ < 0.01, p = 0.963). When adding Group as between-subject variable, we found no significant Group × Dissociation (F_(1,58)_ = 2.34, p = 0.131) or Group × Dissociation × Hemisphere (F_(1,59)_ = 2.06, p = 0.156) interaction. Furthermore, after adding Group, the main effect of Dissociation became non-significant (F_(1,61)_ = 3.53, p = 0.065). However, separate analyses of each group suggested that the association between Dissociation and mean FA in UF was strongest in PTSD participants (F_(1,30)_ = 7.88, p = 0.008; TRC: F_(1,25)_ = 0.80, p = 0.377).

### 6.8 Associations between symptoms of avoidance and CB FA

In contrast to our hypothesis, higher avoidance scores were not associated with lower mean FA in CB (main effect of avoidance: F_(1,61)_ = 3.37, p = 0.071). However, we observed an interaction between Avoidance and Hemisphere interaction effect (F_(1,61)_ = 8.47, p = 0.005), and separate analyses showed that lower right CB FA was associated with higher avoidance scores (F_(1,61)_ = 8.78, p = 0.004), while associations with left CB FA were absent (F_(1,61)_ < 0.01, p = 0.961) (Fig. 3). When entering Group there was no main effect of avoidance (F_(1,61)_ = 1.44, p = 0.289) or Group × Avoidance interaction effect (F_(1,58)_ = 0.01, p = 0.971). However, we observed a significant Group × Avoidance × Hemisphere interaction effect (F_(1,59)_ = 11.255, p = 0.001), with PTSD participants, but not TRC, showing lower right CB FA with higher avoidance scores, while both PTSD participants and TRC did not show any associations between avoidance and left CB FA. Follow-up F-tests for left and right CB separately for TRC and PTSD, indicated that the association between right CB FA and avoidance for PTSD participants was significant (PTSD participants right CB FA; F_(1,30)_ = 7.62, p = 0.009; TRC right CB FA: F_(1,25)_ = 0.19, p = 0.666; PTSD participants left CB FA: F_(1,30)_ = 0.63, p = 0.432; TRC left CB FA: F_(1,25)_ = 1.68, p = 0.21).

## 7 Discussion

We found that treatment seeking male refugees with PTSD differed in WM microstructure relative to trauma-exposed male refugee healthy controls. Confirming our hypotheses, participants with PTSD had lower mean FA values in CB, OF-ST and SLF tract segments, and tract-specific FA reductions scaled positively with having experienced multiple traumatic events. While we confirmed our hypothesis that state measure of dissociation would be positively associated with FA in UF, we neither found between-group differences in FA nor a negative relation between arousal and regional FA in UF. We also did not find the expected negative relationship between avoidance and FA in bilateral CB, but an association of lower right CB FA with higher avoidance scores, specifically in PTSD participants.

### 7.1 Group differences in tract segments FA

Our finding that PTSD was associated with lower FA in CB is in accordance with previous literature of DWI-studies applying both tract-based spatial statistics (TBSS) and tractography based region of interest approaches (Averill et al., 2018, Daniels et al., 2013, O'Doherty et al., 2018, Siehl et al., 2018). The observation that PTSD also was associated with lower AD and higher RD supports the interpretation of decreased integrity (e.g. decreased myelination, axonal density etc.) of CB in PTSD (see also below). The prevailing neurocircuitry model of PTSD implicates altered functional connectivity among mPFC, amygdala, insula and hippocampus in abnormal fear conditioning and fear-potentiated startle, as well as deficits in contextual processing and memory function (Garfinkel et al., 2014, Harnett et al., 2018, Rauch et al., 2000, Shin et al., 2005). Our findings support this model, because microstructural disintegration of the CB has the potential to affect communication between these regions (Bubb et al., 2018, Schmahmann and Pandya, 2006, Von Der Heide et al., 2013).

We also found lower FA and higher RD values in OF-ST fibre tracts in PTSD participants relative to TRC. The functional implications of this finding remain to be clarified. Although the fibre bundles between OF and striatum have not specifically been investigated in PTSD, a decreased integrity of OF-ST fibre tracts may contribute to anhedonia, as anhedonia in PTSD has been linked to reduced task-related functional recruitment of orbitofrontal regions and ventral striatum (Felmingham et al., 2014, Frewen et al., 20112011, Frewen et al., 2010, Olson et al., 2018, Sailer et al., 2008). Of note, we found reduced orbitofrontal ventral striatal activation, when measuring brain activity during a monetary reward paradigm in the same sample population that was investigated in the present study (Uldall et al., 2020b).

In our study, microstructural between-group differences were confined to the medial portions of SLF, subsegment I and II. This is in accordance with previous DWI studies applying TBSS analysis in which PTSD participants had lower FA in regions connected to SLF such as prefrontal lobe, posterior internal capsule, cingulum and tapetum in corpus callosum compared to healthy controls (Fani et al., 2012b; Schuff et al., 2011b). SLF I and II fibres connect the superior parietal region with the frontal lobe supplementary motor areas (SMA) and are likely involved in salience processing (Schmahmann and Pandya, 2006, Seeley et al., 2007), which a meta-analysis of functional MRI studies has shown to be abnormal in PTSD (Hayes et al., 2012). Also, compared to healthy controls, PTSD has been linked to lower resting-state connectivity in several regions including SMA (Hayes et al., 2012).

### 7.2 Interpreting DT metrics in the context of PTSD

Explorative post-hoc examinations of MD, AD and RD measures, suggested that PTSD was associated with lower AD in right CB, left SLF-I and left SLF-II and higher RD in bilateral OF-ST relative to TRC. These findings are in line with the observed lower FA for each tract. The opposite direction of observed differences in AD and RD could be caused by a difference in axonal alignment at a voxel level. At a cellular level, higher RD may also be due to decreased axonal or myelin density. AD reductions have been associated with neuronal degeneration leading to reduced mobility in the cytosol (Dyrby et al., 2018, Novikov et al., 2018).

Several mechanisms reflecting both pathophysiological degeneration and plasticity following experience-dependent plasticity could drive white matter changes in PTSD. Recently, there has been accruing evidence of increased immunological activity of proinflammatory cytokines in PTSD (Bam et al., 2016, Elenkov and Chrousos, 2002, Favrais et al., 2011, Gola et al., 2013, Wang et al., 2017) which, in light of their possibly hypomyelination effect (di Penta et al., 2013, Lin et al., 2005, Popko and Baerwald, 1999), might constitute degenerative factors of WM in PTSD. Moreover, both an increase (Fields, 2015, Fields, 2008, Gibson et al., 2014) and a decrease in neural activity, can shape WM myelination of the adult brain in a bidirectional fashion (Etxeberria et al., 2016, Hines et al., 2015, Korrell et al., 2019, Lazari et al., 2018, Lehmann et al., 2017, Liu et al., 2012, Sinclair et al., 2017). The positive correlation between UF FA and the state measure of dissociation in PTSD participants could indicate such experience-dependent myelination counteracting degenerative effects, as discussed below.

### 7.3 Correlations between FA and immediate symptoms in response to trauma provoking stimuli

In agreement with our prediction, FA in UF reflected inter-individual variations in dissociation symptoms. The higher the individual dissociation score, the higher was FA in left and right UF. This association seemed to be strongest in PTSD participants. To the best of our knowledge no other study has investigated the relationship between state symptoms of dissociation and microstructural properties of WM in UF. Therefore, the robustness of this finding needs to be confirmed in future studies. Regional FA has previously been shown to increase in response to neural activity, e.g. following a 6-week training intervention during which participants learned complex visuo-motor skills (Scholz et al., 2009) or two weeks of working memory training (Takeuchi et al., 2010). For a review of white matter plasticity, see Sampaio-Baptista and Johansen-Berg, 2017. We thus hypothesize that our finding may reflect neural plasticity that couples lasting changes in regional activity in prefrontal cortex with lasting alterations to microstructural properties of white matter structures. Since the UF connects mPFC and limbic regions, we speculate that the observed increase FA in the UF is underpinned by increased mPFC-limbic activity and hence supports the outlined model for dissociation in PTSD (van Huijstee and Vermetten, 2018).

At variance with our hypothesis, high individual avoidance scores were not associated with lower FA in bilateral CB. Instead, there was a significant interaction between hemisphere and individual avoidance scores, with lower right mean FA in CB being associated with higher avoidance scores. Moreover, his relationship was specifically driven by PTSD participants. In agreement with our finding, a recent study found a negative linear relationship in PTSD participants between lifetime measures of avoidance and FA only in the CB of the right hemisphere (O'Doherty et al., 2018). Avoiding memories, people and places that can trigger trauma-related emotional responses is a hallmark of PTSD (Weathers et al., 2015) and are considered an affect regulating strategy (Stiles, 1999), as it reduces the risk of being confronted with painful memories. However, on the long run such avoidance strategy can have unfavourable effects by prohibiting trauma recall in safe therapeutic surroundings and thus hinder effective treatment (Capaldi et al., 2017, Debeer et al., 2013, Ganly et al., 2017, Hermans et al., 2005). Considering that CB plays an important role in episodic memory retrieval (Bubb et al., 2018, Ezzati et al., 2016, Kantarci et al., 2011) and that PTSD is marked by memory dysfunctions (Johnsen and Asbjørnsen, 2008, Scott et al., 2015) one might speculate if avoidance and decreased memory specificity in trauma-affected refugees with PTSD is, in part, mediated by alterations in the microstructure in CB.

### 7.4 Lateralization effects

We found that the group difference in FA in CB was significantly lateralized to the right hemisphere. The negative associations between number of personally experienced traumatizing events and FA measure in CB and OF-ST was also limited to the right hemispheres, as was the negative association between symptoms of avoidance and CB FA. These observations agree with some previous DTI studies on CB in PTSD where asymmetry and a pattern toward right side lateralization also emerged with respect to group differences (Daniels et al., 2013, Siehl et al., 2018). Moreover, several studies have documented a right sided lateralisation in PTSD with smaller volumes of hippocampus (Winter and Irle, 2004) and anterior cingulate cortex (Kitayama et al., 2006) and decreased concentration of N-acetyl aspartate (indicative of low neural density) in hippocampus (Schuff et al., 2001), as compared to healthy controls. Also, right lateralisation of changes in microstructural properties of WM in fibre tracts in relation to negative emotionality has previous been found (Madsen et al., 2012), which is of interest in the context of PTSD where fear is a clinical hallmark. Moreover, in rodents cortisol stress responses mediated by the hypothalamic–pituitaryadrenal (HPA) and PFC have been found to be predominantly regulated by right hemisphere (Cerqueira et al., 2008; Sullivan and Gratton, 2018), which is of relevant in the context of PTSD as the disorder is characterized by abnormal stress regulation (Fragkaki et al., 2016; Pitman et al., 2012). However, more studies are needed to assert the significance and mechanism behind structural lateralization of WM tracts in PTSD.

### 7.5 Methodological considerations

In the current study we employed a relatively new tract segmentation method that provides robust subject specific tract segmentations. The method is automatic and robust to operator-based biases and has recently reported improved performance in comparison with other tractography based methods or registration to common atlas spaces (Wasserthal et al., 2018). The segmentation accounts for complex fiber configurations with a crossing fiber model and performs the segmentation on the fiber direction maps directly rather than on reconstructed streamlines as more conventional tractography methods. Most previous DTI studies of PTSD (Siehl et al., 2018) have used Tract Based Spatial Statistics (TBSS) (Smith et al., 2006) or other registration techniques to common templates to compare diffusivity metrics across subjects. TBSS compares voxels on a white matter skeleton which may not optimally capture inter-individual variations of tract trajectories through white matter and their relation to cortical targets. Moreover, TBSS may exclude functionally relevant white matter from the analysis by projecting the highest neighbouring FA value to the skeleton while the current analysis reflects the whole cross section of the respective tracts. The isolation of tracts on an individual basis, furthermore, gives information regarding tract volume in each participant that can be used as covariate to uncouple effects from variation in macroscopic tract volume, as was done in the present study. This has previously shown necessary to account for partial volume effects in microstructural interpretation of DTI data with both tractography and TBSS (Szczepankiewicz et al., 2013, Takao et al., 2011, Vos et al., 2011).

## 8 Limitations

One important limitation is that the study included male refugees only. Since sex differences in PTSD symptomatology have previously been found (Charak et al., 2014), there may also be important gender differences in the neurobiology of PTSD (Pineles et al., 2017). Therefore, the results are limited to trauma-affected male refugees. The authors strongly encourage a replication study in females and a general prioritization of mixed-sex or female-only studies so that advances in understanding and potential treatment improvements are not gender-exclusive.

Trauma exposure has previous been associated with alterations in WM tracts (Costanzo et al., 2016, Kawashima et al., 2014, O'Doherty et al., 2018) and thus the use of trauma-affected controls only (though less affected than the PTSD participants) in this study may conceal any potential effects of traumatic events per se. Furthermore, as we did not perform the CAPS interview in the TRC group, we were limited to a binary PTSD outcome (PTSD versus TRC) instead of assessing PTSD as a continuous outcome using CAPS (sub)scores. Thus, we were not able to reveal possible WM changes in e.g., participants with subthreshold PTSD. Therefore, the results concern WM alterations associated with the transition from being trauma-affected to clinical PTSD. Because there was a high rate of antidepressant medication use in our PTSD sample and antidepressants have been shown to affect the microstructure of WM (Steffens et al., 2008), our results cannot be generalized to a medication-free PTSD population. Moreover, as depression was present in 86% of the PTSD sample, our results should be interpreted with caution as they may have limited validity in other samples than trauma-affected male refugees with PTSD and secondary depression. Generally, depression is diagnosed in approximately 50% of PTSD cases and it is appropriate to consider depression and PTSD to emerge simultaneously as two facets of a general posttraumatic psychopathology (Flory and Yehuda, 2015, O’Donnell et al., 2004). Consequently, our results can be generalized to a large, clinically relevant, population of treatment seeking refugees with PTSD. However, there is clearly a need for exploring a wider spectrum of PTSD populations that spans different symptom dimensions. Since we used interpreters there is a risk of miscommunication though it was not the impression that this has resulted in any clinically relevant information being lost. The prevalence of mTBI was evenly distributed among the TRC and PTSD groups and was included as covariates in all analysis. However, since mTBI is associated with altered white-matter microstructure (Narayana, 2017) and in one recent study has been shown to account for the differences in FA between two groups with and without PTSD (Klimova et al., 2019), it is questionable whether our findings extends to populations with PTSD with no history of mTBI. Finally, longitudinal studies are needed to assert if biological dispositions to WM pathology are involved in the causation of PTSD, or vice versa.

Methodologically, conventional DWI data and the interpretation of DT metrics are limited in specificity. Future studies could consider novel diffusion encoding techniques. Multidimensional diffusion encoding (Lundell and Lasič, 2020) could for instance be used to decompose variation in cell shape, size and orientation and diffusion encoding, which combined with spectroscopy may enable the separation of microstructural changes in glial and neuronal cell pools (Lundell et al., 2020).

## 9 Conclusion

We found that PTSD is associated with compromised regional changes of WM microstructure of WM in CB, OF-ST and SLF, segments I and II, and that state symptoms of dissociation and avoidance were associated with changes in microstructural properties of UF and CB. Our microstructural findings support a neuro-circuit model of PTSD that emphasizes dysregulation of hippocampus, amygdala and insula by mPFC in relation to a heterogeneous spectrum of altered behaviour including contextual processing deficits, exaggerated fear and symptoms of avoidance and dissociation. The findings are also compatible with disturbances in the reward circuitry involving striatum, and symptoms of anhedonia in PTSD.

## CRediT authorship contribution statement

**Sigurd Wiingaard Uldall:** Conceptualization, Methodology, Formal analysis, Investigation, Writing – original draft, Writing – review & editing, Visualization. **Henrik Lundell:** Methodology, Formal analysis, Investigation, Writing – original draft, Writing – review & editing, Software, Visualization. **William F.C. Baaré:** Methodology, Formal analysis, Investigation, Writing – review & editing, Supervision. **Hartwig Roman Siebner:** Conceptualization, Methodology, Writing – review & editing, Supervision. **Egill Rostrup:** Conceptualization, Methodology, Writing – review & editing, Supervision. **Jessica Carlsson:** Conceptualization, Methodology, Investigation, Writing – review & editing, Supervision.

## Declaration of Competing Interest

The authors declare the following financial interests/personal relationships which may be considered as potential competing interests: Hartwig Roman Siebner has received honoraria as speaker from Sanofi Genzyme, Denmark and Novartis, Denmark, as consultant from Sanofi Genzyme, Denmark, Lophora, Denmark, and Lundbeck AS, Denmark, and as editor-in-chief (Neuroimage Clinical) and senior editor (NeuroImage) from Elsevier Publishers, Amsterdam, The Netherlands. He has received royalties as book editor from Springer Publishers, Stuttgart, Germany and from Gyldendal Publishers, Copenhagen, Denmark. Henrik Lundell has received funding from the European Research Council (ERC) under the European Union’s Horizon 2020 research and innovation programme (grant agreement No 804746). HRS holds a 5-year professorship in precision medicine at the Faculty of Health Sciences and Medicine, University of Copenhagen which is sponsored by the Lundbeck Foundation (Grant Nr. R186-2015-2138).

## Figures and Tables

**Supplementary figure 4:**